# IGF2BP2-modified circular RNA circCHD7 promotes endometrial cancer progression via stabilizing PDGFRB and activating JAK/STAT signaling pathway

**DOI:** 10.1038/s41417-024-00781-9

**Published:** 2024-05-22

**Authors:** Rui Shi, Rong Zhao, Yan Shen, Sitian Wei, Tangansu Zhang, Jun Zhang, Wan Shu, Shuangshuang Cheng, Hua Teng, Hongbo Wang

**Affiliations:** 1 Department of Obstetrics and Gynecology, Union Hospital, Tongji Medical College, Huazhong University of Science and Technology, Wuhan, Hubei 430022 PR China; 2 Health Management Center, Union Hospital, Tongji Medical College, Huazhong University of Science and Technology, Wuhan, Hubei 430022 PR China

**Keywords:** Oncogenes, Tumour biomarkers

## Abstract

Circular RNAs (circRNAs) represent a class of covalently closed, single-stranded RNAs and have been linked to cancer progression. N6-methyladenosine (m^6^A) methylation is a ubiquitous RNA modification in cancer cells. Increasing evidence suggests that m^6^A can mediate the effects of circRNAs in cancer biology. In contrast, the post-transcriptional systems of m^6^A and circRNA in the progression of endometrial cancer (EC) remain obscure. The current study identified a novel circRNA with m^6^A modification, hsa_circ_0084582 (circCHD7), which was upregulated in EC tissues. Functionally, circCHD7 was found to promote the proliferation of EC cells. Mechanistically, circCHD7 interacted with insulin-like growth factor 2 mRNA-binding protein (IGF2BP2) to amplify its enrichment. Moreover, circCHD7 increased the mRNA stability of platelet-derived growth factor receptor beta (PDGFRB) in an m^6^A-dependent manner, thereby enhancing its expression. In addition, the circCHD7/IGF2BP2/PDGFRB axis activated the JAK/STAT signaling pathway and promoted EC cell proliferation. In conclusion, these findings provide new insights into the regulation of circRNA-mediated m^6^A modification, and the new “circCHD7-PDGFRB” model of regulation offers new perspectives on circCHD7 as a potential target for EC therapy.

## Introduction

Endometrial cancer (EC) is one of the most common gynecological malignancies, presenting an increasing incidence and disease-related mortality worldwide [1]. The overall incidence has increased by 132% over the last 30 years, with 417,000 new cases of endometrial cancer diagnosed worldwide in 2020 [2]. Early-stage EC is treated predominantly by surgery, while advanced and hormone-non-dependent ECs are mostly treated with carboplatin/paclitaxel [3]. However, advanced, recurrent, or metastatic EC tumors demonstrate poor clinical efficacy, highlighting the importance of elucidating the underlying molecular mechanisms and identifying precise predictive biomarkers of EC are crucial. Such biomarkers could improve early diagnosis and optimal treatment of EC, and be applied for monitoring cancer recurrence and metastasis.

As a class of covalent closed-loop RNA molecules, circRNAs are generated by reverse splicing of pre-mRNA transcripts without a 5’ cap and 3’ polyadenylate tail [4–6]. Previous studies have shown that circRNAs can adopt various forms and are involved in potential mechanisms of cellular physiology, including sponge miRNAs, binding to proteins, translation into peptides and proteins, and acting as a transcriptional or translational regulator [7]. Dysregulated circRNAs regulate proliferation, metastasis, angiogenesis, drug resistance, metabolism, and immune response in various cancers [8, 9]. Some circRNAs can neither be adsorbed by miRNA sponges nor translate peptides, but still carry out important functions through physical interactions with proteins [10, 11]. In oral squamous cell carcinoma, circFNDC3B enhances angiogenesis by regulating ubiquitination of the RNA-binding protein FUS and deubiquitination of HIF1A via the E3 ligase MDM2, thereby promoting VEGFA transcription [12]. In gastric cancer cells, a complex containing overexpressed circIPO7 blocks the caprin-1-G3BP1 interaction, dissociating caprin-1 and its target mRNAs (EGFR and mTOR) from the ribosome, leading to inhibition of their translation and consequent inactivation of the PI3K/AKT/mTOR pathway [13]. Previous studies have reported that circRNAs in EC mainly function as miRNA sponges, but only a few studies have investigated protein binding and encoding peptides. Hence, the expression pattern and function of circRNAs in EC remains unelucidated.

N6-methyladenosine (m^6^A) modification has gained increasing interest as a new area of epigenetic regulation with multiple functions [14]. M^6^A-dependent mRNA modification is an important regulator of mRNA and non-coding RNA production and function. It plays an essential role in cancer development and various biological processes by regulating RNA stability, splicing, translocation, and nuclear localization [15–17]. The m^6^A modification is reversible and dynamic and is regulated by three elements, namely methyltransferases (writers), demethylases (erasers), and binding proteins (readers) [18–20]. The interaction between m^6^A modifications and circRNAs plays a crucial role in a variety of cancer and disease processes, as evidenced by their influence on tumor growth, invasion, metastasis, colony formation, chemoresistance, and immune response. IGF2BP2, recently reported as an m^6^A binding protein, promotes AML development and leukemic stem cell/initiating cell self-renewal by regulating the expression of key targets in the glutamine metabolic pathway (e. g. MYC, GPT2, and SLC1A5) in an m^6^A-dependent manner [21]. Furthermore, the E6/E7 protein regulates MYC mRNA m^6^A modification via IGF2BP2 to promote aerobic glycolysis, proliferation, and metastasis of cervical cancer cells [22]. Increasingly, circRNAs have been shown to mediate cancer progression by regulating m^6^A modifications. In addition, circMYO1C targets the m^6^A site of PD-L1 mRNA by binding to IGF2BP2, enhancing its stability and accelerating PDAC immune escape [23]. circARHGAP29 interacts with IGF2BP2 to improve lactate dehydrogenase A (LDHA) mRNA stability, thereby enhancing tumor cell glycolysis; the molecule provides a promising therapeutic target for docetaxel-resistant prostate cancer [24]. However, the mechanism underlying the regulatory effects of circRNAs on endometrial cancer progression via IGF2BP2 remains unknown.

This study focused on up-regulated circRNAs based on high-throughput sequencing data from human EC and normal endometrial tissues [25] and identified hsa_circ_0084582 (circCHD7), a novel circRNA produced by cyclization of exon 2 of the CHD7 gene and subjected to m^6^A modification. Functionally, circCHD7 promotes EC cell proliferation and inhibits apoptosis in vitro and in vivo. Mechanistically, circCHD7 binds to IGF2BP2 and promotes the expression of the downstream target gene platelet-derived growth factor receptor beta (PDGFRB) in an m^6^A-dependent manner by increasing mRNA stability, thereby inhibiting EC cell apoptosis via the JAK/STAT pathway. Collectively, this study depicts a novel mechanism of circCHD7/m^6^A/IGF2BP2/PDGFRB and highlights its role in the regulation of EC cell proliferation.

## Methods

### Human tissues sample and databases

Altogether, 15 human normal endometrial tissues and 41 EC tissues were collected from surgical patients at the Union Hospital of Tongji Medical College, Huazhong University of Science and Technology (Wuhan, China) from January 2018 to January 2021. The pathological types of all tissue specimens were confirmed by pathologists. Detailed clinical characteristics and histopathological types of the patients are described in Table 1. All tissue samples were stored in liquid nitrogen until used. Patients read and signed an informed consent form prior to the operation. The Institutional Review Board of Tongji Medical College, Huazhong University of Science and Technology approved the study.

**Table 1:** Clinicopathologic features of 41 patients with EC and the expressions of circCHD7.

			CircCHD7 expression				
Variables	Group	Cases	Low	%	High	%	*p*
Age of surgery	<60	18	13	52	5	31.3	0.163
	≥60	23	12	48	11	68.7	
Pathologic stage	Stage I	18	14	56	4	25	0.047
	Stage II	10	7	28	3	18.8	
	Stage III	8	3	12	5	31.3	
	Stage IV	5	1	4	4	25	
Pathologic type	Endometrioid	32	21	84	11	68.8	0.221
	Non-endometrioid	9	4	16	5	31.2	
Grade	G1	10	2	8	8	50	0.024
	G2	10	5	20	5	20	
	G3	21	18	72	3	30	
Lymphatic metastasis	Absent	32	23	85.2	9	64.3	0.157
	Present	9	4	14.8	5	35.7	

### Quantitative real-time polymerase chain reaction (qRT-PCR)

According to the instructions, total RNA was extracted from cells or fresh tissues with TRizol (#9108, RNAiso Plus, Takara, Japan). qRT-PCR was performed by Real-Time PCR Kit (Takara, Japan) and the Biosystem StepOne Plus PCR System (ABI). circRNA and mRNA levels were standardized by GAPDH, and RNA expression levels were calculated by the 2-ΔΔCT method. The primers used are listed in Supplementary Table 1.

### Cell culture

EC cell lines included Ishikawa, KLE, RL-952, HEC-1A, and HEC-1B, which were purchased from the American Type Culture Collection (ATCC; Manassas, VA). Ishikawa cells were cultured in DMEM/F12 medium (#11330032, Gibco) supplemented with 10% fetal bovine serum (#10099141, Gibco) and 1% streptomycin and penicillin (#PYG0016, Bosterbio, USA). HEB-1C cells were cultured in MEM medium (#41500034, Gibco) with 10% fetal bovine serum (#10099141, Gibco) and 1% streptomycin and penicillin (#PYG0016, Bosterbio, USA). All of them were cultured in the 5% CO_2_, 37 °C incubator.

### Transfection

Lentivirus vector containing short hairpin RNA (shRNA) of circCHD7 were purchased from Genomeditech (Shanghai, China) (Supplementary Table 1). IGF2BP2 three short hairpin RNAs (shRNA), METTL3 short hairpin RNA (shRNA), and negative control shRNA were designed and synthesized by GenChem (Shanghai, China) (Supplementary Table 1). The human PDGFRB cDNA were synthesized by TSINGKE, which were cloned into p3XFLAG-CMV-10 vector (Sigma-Aldrich) to construct overexpression plasmid (Supplementary Table 1). Lentiviral transfection of cells was performed when cell integration reached 20–30%. Adherent cells are fused to about 60% for transfection of plasmids. We established stable cell lines using a lentivirus-mediated delivery system at a multiplicity of infection (MOI) of 30 using polyglutamine. Cells were selected using 2 μg/mL puromycin for 48 h. To construct the stably transfected cell lines, plasmids(2 μg/gel) were transfected into cells by using Lipofectamine 3000 (Life Technologies) according to the manufacturer’s instructions and then the cells were filtered with G418 (Invitrogen) for 4–6 weeks.

### RNase R treatment and actinomycin D

2 μg of total RNA was incubated with or without 3 U/μg of RNase for 30 min at 37 °C. The total RNA was obtained for the actinomycin D treatment assay after treating cells with 5 μg/mL of actinomycin D for 0 h, 4 h, 8 h, 12 h and 24 h. After cells were treated with RNAase and actinomycin D, the qRT-PCR procedure was operated as described previously to evaluate changes in expression of circCHD7 and CHD7 mRNA.

### Nuclear-cytoplasmic fraction

Isolation of cytoplasmic and nuclear RNA was performed with the Nucleoplasmic RNA Purification Kit (#21000, Norgenbiotek, Canada). Cells were washed twice with pre-cooled PBS, 200 µl of pre-cooled lysis buffer J was added and placed on ice for 5 min. The cell lysate was centrifuged at 12,000 rpm for 10 min at 4 °C to separate the cytoplasmic RNA containing fraction and the nuclear RNA fraction. The cytoplasmic and nuclear solutions were washed sequentially by vortexed with Buffer K and 100% ethanol and subsequently transferred to a centrifuge column for centrifugation. The centrifugation column was washed three times with Washing Solution A and then the RNA was lysed with 50 µl of Elution Buffer E to obtain the RNA solution.

### Fluorescence in situ hybridization (FISH) and RNA FISH-immunofluorescence microscopy

Cy3-labeled circCHD7, human U6 and human 18 S probes were designed and synthesized by RiboBioCo. Ltd. (Guangzhou, China) (Supplementary Table 1). FISH assays were conducted by using the RiBoTM Fluorescence in Situ Hybridization Kit (#C10910, RiboBioCo Ltd., Guangzhou, China) according to the instructions. Cells (1 × 10^4 / well) were planted in 24-well plates and cultured overnight in the cell incubator prior to the FISH assay. After incubation in the pre-hybridization solution for 30 min at 37 °C, cells were hybridized with the fluorescent probe overnight at 37 °C shielded from light. The subsequent day, cells were rinsed three times with 4× / 2× / 1× SCC solution for 5 min each followed by DAPI staining for 10 min at 42 °C under light-protected conditions. Cells were eventually observed with confocal microscopy at 400 × magnification.

RNA FISH-immunofluorescence microscopy was to detect co-localization of circCHD7 and IGF2BP2 proteins. Ishikawa cells and HEC-1B cells were fixed, permeabilityed and pre-hybridized. Subsequently, hybridization was performed overnight at 37 °C protected from light using the Cy-3-labelled circCHD7 probe, and the cells were subsequently washed utilizing SSC buffer at 42 °C. Cells were then hatched with primary antibody (1:1000) for 1 h at room temperature, followed by reaction with Alexa Fluor 594 or 488-conjugated secondary antibody (1:1000 dilution; Cell Signaling Technology) and DAPI for 30 min. Images were obtained using confocal microscopy.

### Cell counting Kit-8 Assay and colony formation assay

The Cell Counting Kit-8 Assay (#34302, CCK8, Bimake, USA) was used to detect cell proliferation according to the manufacturer’s instructions. After treatment, cells (5 × 10^3 / well) were planted in 96-well plates and incubated for respective time periods (0 h, 24 h, 48 h, 72 h, 96 h). 10 μL of CCK8 solution was added to each well and incubated at 37 °C for 1 h. The absorbance at 450 nm was captured using an automated microplate reader (BioTek, VT, USA).

For the colony formation assay, transfected cells were inoculated into 6-well plates at a density of 600 cells per well and maintained in DMEM/F12 or MEM medium containing 10% FBS for 2 weeks. Cells were washed twice with PBS, fixed in methanol for 10 min and stained with 0.1% crystal violet for 30 min, then colonies were imaged and counted.

### EdU assay

Cells (1 × 10^^4^ cells / well) were transplanted in 96-well plates and incubated overnight at 37 °C with 5% CO_2_ thermostat prior to EdU (5-ethynyl-2’-deoxyuridine) assay. EdU assays were performed using the EdU kit (#C0071S, Beyotime, shanghai) according to the instructions. EdU solution was prepared as 1:1000 EdU medium. Cells were incubated in pre-prepared 96-well plates with 50 µl / well of EdU medium for 2 h at 37 °C and fixed in 4% paraformaldehyde for 15 min. Cells were incubated with 50 ul/well of click reaction solution for 30 min at room temperature protected from light and then Hochst33342 stained for 10 min. Proliferating cells were imaged with a 200 × microscope and five isolated areas were photographed for counting purposes.

### Migration assays

Cell migration assays were performed in 24-well plates. 600 µl of medium containing 20% fetal bovine serum was added to the lower part of the chamber (#3422, Corning, USA) and 200 µl of serum-free medium containing 8 × 10^^4^ cells were added to the upper part of the chamber. Cells were incubated at 37 °C for 24 h and penetrated from the top to the bottom of the chamber membrane (8 μm pore size). Cells that had penetrated the chamber membrane were fixed in 4% paraformaldehyde for 15 min and stained with 0.1% crystalline violet for 30 min, then were imaged under a 100 × microscope and four separate fields were taken for counting.

### Flow cytometry apoptosis assay

Ishikawa and HEC-1B cells were harvested, stained with FITC Annexin V and propidium iodide (PI) (BD Pharmingen) and subsequently analysed for apoptosis using flow cytometry (Becton Dickinson). PI-negative and FITC Annexin V-positive cells were identified at earlier stage of apoptosis, while cells in advanced stage of apoptosis or already dead cells were double positive for PI and FITC annexin V. Results were analyzed with FlowJo software.

### RNA pulldown

The biotinylated-circCHD7 probe was synthesized by Sangon Biotech (Shanghai, China) (Supplementary Table 1). The sequence of the circCHD7 antisense probe is complementary to the reverse copy node of circCHD7 only. Briefly, 2 × 10^7^ EC cells were collected, lysed, sonicated, and centrifuged. 50 µl of cell lysate was obtained as input and the remainder was divided equally and mixed with biotin-labelled antisense probes or sense probes and incubated for 2 h at room temperature. The cell lysate containing the probe was then incubated with streptavidin magnetic beads (#HY-K0208, MedChemExpress, USA) for 1 h at room temperature. The magnetic beads were then washed thoroughly with lysis buffer at least three times. The RNA-protein binding mixture was boiled in SDS buffer and the bound proteins were detected by western blot assay and mass spectrometry (MS).

### Methylated RNA immunoprecipitation-qPCR (MeRIP-qPCR)

Total RNA was isolated with TRIzol reagent (Invitrogen). methylated RNA immunoprecipitation (MeRIP) was performed using the Magna MeRIP m^6^A kit (Millipore, Billerica, MA, USA). In brief, an anti-m^6^A antibody was attached to protein A/G beads and then incubated with fragmented RNA. The enriched RNA was purified and analyzed by qRT-PCR.

### RNA immunoprecipitation

RNA-binding protein immunoprecipitation (RIP) assays were performed using the EZ-Magna RIP kit (#17-704, Millipore, Burlington, MA, USA) in accordance with the instructions. The purpose of RIP was to extract and identify protein-bound RNA. Cells (1 × 10^7) were added to the lysis solution and lysed on ice for 5 min. The cells were then centrifuged and the supernatant extracted. After incubation with 5 µg of the target antibody or IgG antibody for 30 min, the magnetic beads were combined with 100 µl of cell lysate supernatant, respectively, and incubated overnight at 4 °C, with rotation. The magnetic bead protein RNA complexes were washed, and then RNA was extracted and subjected to qRT-PCR.

### Western blot assays

Total cell or tissue protein was extracted using RIPA lysate (#P0013B, Beyotime, Shanghai, China) and protein concentration was measured using the BCA Protein Assay Kit (#C503021, Singon Biotech, Shanghai, China). Total protein (20 µg) was added to 10% or 12.5% SDS-PAGE gels for gel electrophoresis, followed by transmigration onto polyvinylidene difluoride membranes. After blocking with 5% skimmed milk for 1 h at room temperature, the membranes were incubated with primary antibody overnight at 4 °C. Membranes were washed three times with TBST buffer and incubated with goat anti-rabbit or goat anti-mouse secondary antibodies for 1 h at room temperature. Finally, imaging was performed with The ChemiDoc MP (Bio-Rad, USA). Primary antibodies are listed as follows: IGF2BP2 (1:1000, # 11601-1-AP, Proteintech Group, INC., USA), PDGFRB (1:800, # 13449-1-A, ProteintechGroup, INC., USA), Bcl2 (1:1000, # A0208, ABclonal, Wuhan, China), Bax (1:1000, # A19684, ABclonal, Wuhan, China), METTL3 (1:1000, # A19079, ABclonal, Wuhan, China), STAT3 (1:1000, #9139, Cell Signaling Technology, USA), p-STAT3 (1:1000, #9145, Cell Signaling Technology, USA), JAK2 (1:1000, #3344, Cell Signaling Technology, USA), p-JAK2 (1:1000, #74129, Cell Signaling Technology, USA), GAPDH (1:20000, #AC002, ABclonal, Wuhan, China); secondary antibodies are as follows: HRP Goat Anti-Rabbit IgG (1:8000, #AS014, ABclonal, Wuhan, China), HRP Goat Anti-Mouse IgG (1:8000, #AS003, ABclonal, Wuhan, China), China).

### Silver staining and Mass spectrometry analysis

After electrophoresis at 120 V for 75 min, the gel was stained using the Rapid Silver Staining Kit (P0017S, Beyotime) according to the manufacturer’s instructions. Protein profiling was performed by SpecAlly Life Technology Co. (Wuhan, China). In brief, immobilised magnetic bead-bound complexes were washed and digested with sequencing grade modified trypsin. After extraction and purification, peptide samples were identified by mass spectrometry (Q Exactive, Thermo Finnigan, US). The raw mass spectrometry data obtained were retrieved using MaxQuant software (v1.6.2.10). The human reference proteome database of UniProt was used.

### RNA-sequence

To explore in more depth how circCHD7 regulates gene expression in EC cells, RNA-seq was performed in Ishikawa cell cells stably expressing circCHD7. Total RNA was isolated from cultured cells using TRIzol and then sequenced using the NovaSeq 6000 platform (Illumina, USA). RNA-seq was performed as previously reported [26]. KEGG pathway analysis was performed on differentially expressed genes from RNA-seq (|log2(FC) | > 2, *P* < 0.05).

### Hematoxylin-eosin staining

Dewaxing: xylene I and II for 10 min apiece. Dehydration: 100% (I and II), 90%, 80% and 70% alcohol for 5 min each and rinsed 3 times under running water for 5 min each. Staining: sections were stained for 5 min using hematoxylin and rinsed under running water. Acetic acid fractionation for 1 min and rinsing of sections under flowing water. Eosin staining for 1 min and rinsing the slides under running water. 70%, 80%, 90%, and 100% alcohol for 10 s each and xylene for 1 min to dehydrate the slides. Slides were dried spontaneously and sealed with drops of neutral glue.

### Immunohistochemistry (IHC)

Paraffin sections of tumour tissue were dewaxed and dehydrated, hydrated and repaired with citric acid. After peroxidase blockade, tissue sections were incubated overnight at 4 °C with primary antibody. Slides were washed with PBS and incubated with secondary antibody for 30 min at 37 °C. Sections were finally sealed and observed with the microscope. Staining intensity was categorized as negative (score = 0), weak (score = 1), moderate (score = 2) and strong (score = 3); the quantities of positively stained cells were 0–5%, 5–25%, 26–50%, 51–75% and 76–100% respectively. The ultimate staining score was the multiplication of staining intensity and the number of positively stained cell products. Antibodies: IGF2BP2 (1:2000, # 11601-1-AP, Proteintech Group, INC., USA), PDGFRB (1:1000, # 13449-1-A, Proteintech Group, INC., USA), Bcl2 (1:1000, # A0208, ABclonal, Wuhan, China), Bax (1:1000, # A19684, ABclonal, Wuhan, China).

### In vivo tumor xenografts

Animal experiments were supported and approved by the Animal Ethics Committee of Tongji Medical College, Huazhong University of Science and Technology (NO.3722). 6-week-old female BALB/c nude mice were purchased from ShuLaiBao Biotechnology Company (Wuhan, China) and Ishikawa cells (4 × 10^^6^ per mouse) were injected subcutaneously into the back of the mice, after random grouping (6 per group) to conform to a normal or approximately normal distribution. Mice were housed in specific pathogen-free animal houses for 28 days. The subcutaneous tumour sizes in the mice were measured each week. At the end of the experiment (after 4 weeks), tumour sizes were measured and the mice were sacrificed, the tumours removed and weighed. The tumour volume was calculated as: a × b^^2^, where a represented the longest diameter of the tumour and b was the length of the diameter perpendicular to a. For animal studies, the operation should obey blinding.

### Statistical analysis

All statistical analyses were performed with GraphPad Prism v. 8.01 (GraphPad Software, La Jolla, CA, USA). Student’s t-test was applied to compare the values of the test and control groups. For multiple comparisons, differences were analyzed by one-way ANOVA and Turkish post hoc test. *p* < 0.05 was considered statistically significant (*p < 0.05; **p < 0.01; ***p < 0.001; ****p < 0.0001). The variance is similar between the groups that are being statistically compared.

## Results

### Characterization of circCHD7 in EC

**Fig. 1:**
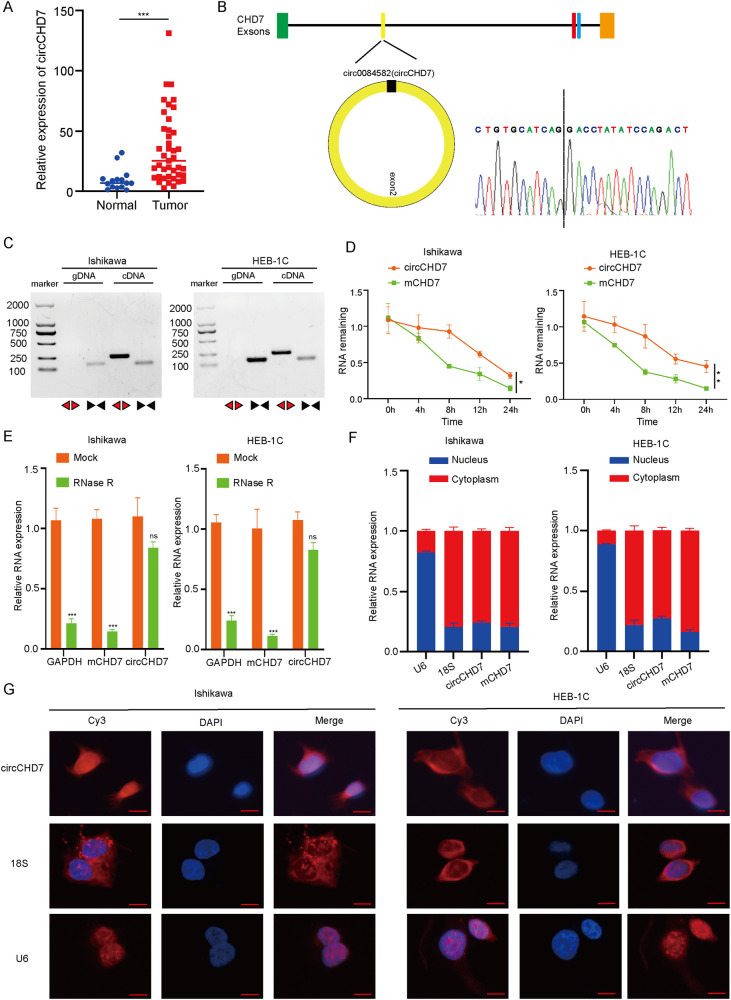
Identification of circCHD7 and detection of its distribution. **A** qRT-PCR analysis showed the expression levels of circCHD7 in EC samples and normal endometrial tissues. **B** The diagram shows the formation of circCHD7. The reverse splice site of circCHD7 was identified by Sanger sequencing. **C** RT-PCR assays using divergent and convergent primers showed that circCHD7 was amplified from cDNA or gDNA of Ishikawa and HEC-1B cell lines. **D**, **E** qRT-PCR assays were performed to detect the stability of circCHD7 when treated with actinomycin D or RNase R. **F**, **G** Nucleoplasmic separation experiment and RNA FISH assay demonstrated the plasmatic localization of circCHD7 in Ishikawa and HEC-1B cells, in which nucleoli were stained with DAPI and circCHD7, U6 and 18 S were stained red by cy3. Scale bar: 100 μm, *P < 0.05; **P < 0.01; ***P < 0.001.

To identify circRNAs that promote EC progression, the differential circRNA data in EC were extracted from the analysis of EC tissue samples by Yongchao Dou et al. [25]. Subsequently, the differential circRNAs were analyzed using R software with the parameters fold change ≥ 1.5 and padj ≤ 0.05. Among the differential circRNAs, 483 circRNAs were down-regulated and 10 were up-regulated (Supplementary Table 2). To determine the presence of m^6^A modification in circRNAs, the above 10 up-regulated circRNAs were analyzed on the SRAMP website (http://www.cuilab.cn/sramp), predicting an abundance of m^6^A modification sites in hsa_circ_0084582 (Supplementary Fig. 1A, B). This enrichment was further investigated after knocking down METTL3 (Supplementary Fig. 1D). Moreover, the impact of METTL3 knockdown on the stability of circCHD7 was explored in Ishikawa cells and HEC-1B cells by actinomycin D exposure for 0, 4, or 8 h. circCHD7 showed decreased stability in Ishikawa cells and HEC-1B cells following METTL3 knockdown (Supplementary Fig. 1E). Meanwhile, the qRT-PCR experiments indicated that the circCHD7 expression levels were significantly decreased in Ishikawa cells and HEC-1B cells with METTL3 knockdown (Supplementary Fig. 1F). Furthermore, the MeRIP-PCR results revealed significantly increased circCHD7 levels in m6A antibody-immunoprecipitated RNA compared to IgG-precipitated RNA (Supplementary Fig. 1G). However, the stability of circCHD7 in endometrial cancer cells was not significantly affected by histone deacetylase inhibitors (5-aza-dc, RGFP966, and SAHA) (Supplementary Fig. 1H). Collectively, these findings indicated the presence of m6A modification sites on the circCHD7 sequence. The qRT-PCR results were validated in the clinical specimens, comprising 15 normal endometrium tissue samples and 41 EC tissue samples, showing consistent results with the previous analysis (Fig. 1A). According to the circBase website (http://www.circbase.org/), hsa_circ_0084582(circCHD7) originated from the exon 2 region within the CHD7 locus and contained 1839 nucleotides (nt), whose backsplice junction sequence was amplified with divergent primers and validated by Sanger sequence (Fig. 1B). To investigate the biological functions of circCHD7 in EC cell lines, qRT-PCR was performed to verify its expression levels in different EC cell lines. The results suggested relatively high circCHD7 expression in Ishikawa cells and HEC-1B cells (Supplementary Fig. 1C). Therefore, Ishikawa cells and HEB-1C cells were selected for subsequent experiments. The cDNA and genomic DNA (gDNA) were amplified using convergent and divergent primers, respectively. Additionally, gel electrophoresis revealed that circCHD7 could be amplified by divergent primers in cDNA but not in gDNA (Fig. 1C). Subsequently, the loop characteristic of circCHD7, circCHD7, and linear mRNA CHD7 was explored by treating with RNase R exonuclease and the transcriptional repressor actinomycin D. The results revealed that circCHD7 was more resistant to both actinomycin D (Fig. 1D) and RNase R (Fig. 1E) compared to mRNA CHD7. To investigate the mechanism of circCHD7 more comprehensively, a cytoplasmic and nuclear RNA isolation and FISH assay were utilized to detect the subcellular localization of circCHD7. The results indicated that circCHD7 was mainly distributed in the cytoplasm of EC cells (Fig. 1F).

In conclusion, these findings identified circCHD7 as a bona fide circRNA, which was regulated by m^6^A modifications, predominantly distributed in the cytoplasm, and significantly upregulated in EC cells.

### CircCHD7 enhanced the proliferative activity of EC cells in vitro

**Fig. 2:**
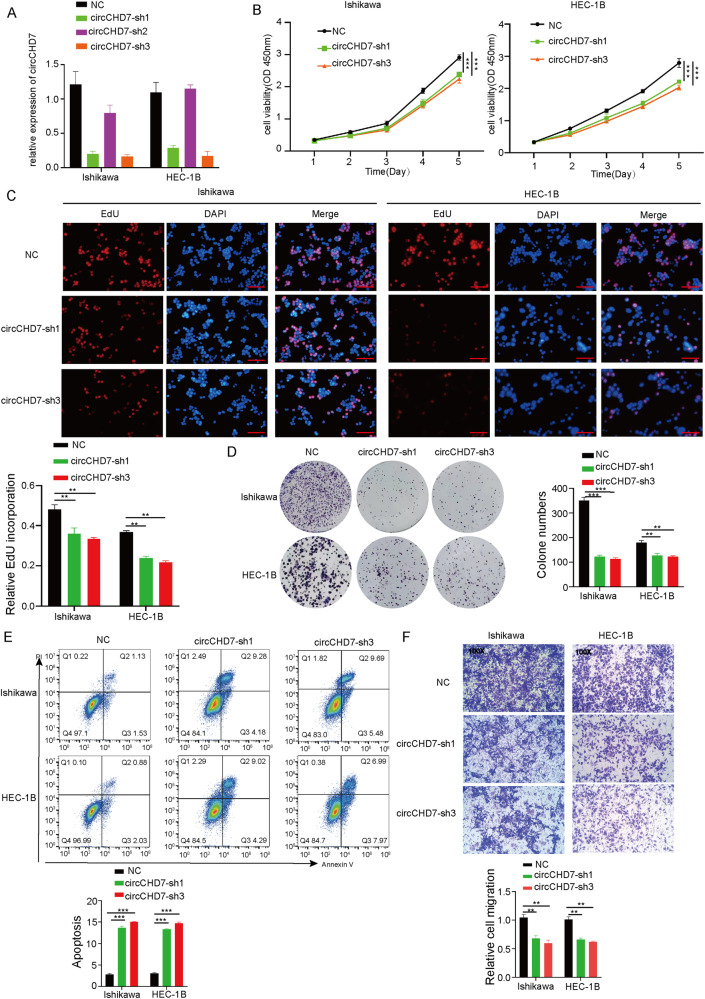
CircCHD7 enhanced the proliferative activity of EC cells in vitro. **A** The expression levels of circCHD7 in Ishikawa and HEC-1B cells transfected with circCHD7-sh1, circCHD7-sh2, circCHD7-sh3, and the corresponding control were detected by qRT-PCR. **B** Viability of Ishikawa cells and HEC-1B cells was assessed at different times using CCK8 reagent. **C** DNA synthesis assessed using EdU assay in indicated cells. **D** Colony formation assay to detect cells proliferation. **E** Flow cytometry assay revealed the rate of apoptosis in Ishikawa and HEC-1B cells transfected with circCHD7-sh1, circCHD7-sh3 and control plasmid. **F** Transwell assay of Ishikawa and HEC-1B cells to detect cell migration ability. Scale bar: 100 μm, *P < 0.05; **P < 0.01; ***P < 0.001.

Nonetheless, the role of circCHD7 in EC progression remains unclear. Therefore, circCHD7 was stably silenced with lentivirus-pack a shRNA plasmids in Ishikawa cells and HEB-1C cells (NC, circCHD7-sh1, circCHD7-sh2, and circCHD7-sh3). The results of the qRT-PCR analysis revealed that circCHD7-sh1 and circCHD7-sh3 significantly downregulated the expression of circCHD7 compared to sh-negative controls (NC) (Fig. 2A). However, there was no significant change on linear CHD7 mRNA level (Supplementary Fig. 1I). To verify the specificity of circCHD7 fluorescence signaling, the results of FISH experiments suggested that compared with the control group, the fluorescence intensity was significantly weakened in the circCHD7-sh1 and circCHD7-sh3 groups, whereas there was no significant alteration in the circCHD7-sh2 group (Supplementary Fig. 3G). The CCK-8, EdU incorporation, and colony formation assays indicated that circCHD7 knockdown impaired the proliferation ability of Ishikawa cells and HEC-1B cells were impaired followed by circCHD7 knockdown (Fig. 2B–D). Moreover, the results of cell flow cytometry experiments suggested increased apoptosis of Ishikawa cells and HEC-1B cells following the knockdown of circCHD7 (Fig. 2E). The migration assays revealed that silencing circCHD7 could reduce the invasion of EC cells (Fig. 2F). Taken together, these results indicated that circCHD7 evidently enhances the proliferation of EC cells.

### CircCHD7 interacts with m^6^A reader IGF2BP2 protein in EC cells

**Fig. 3:**
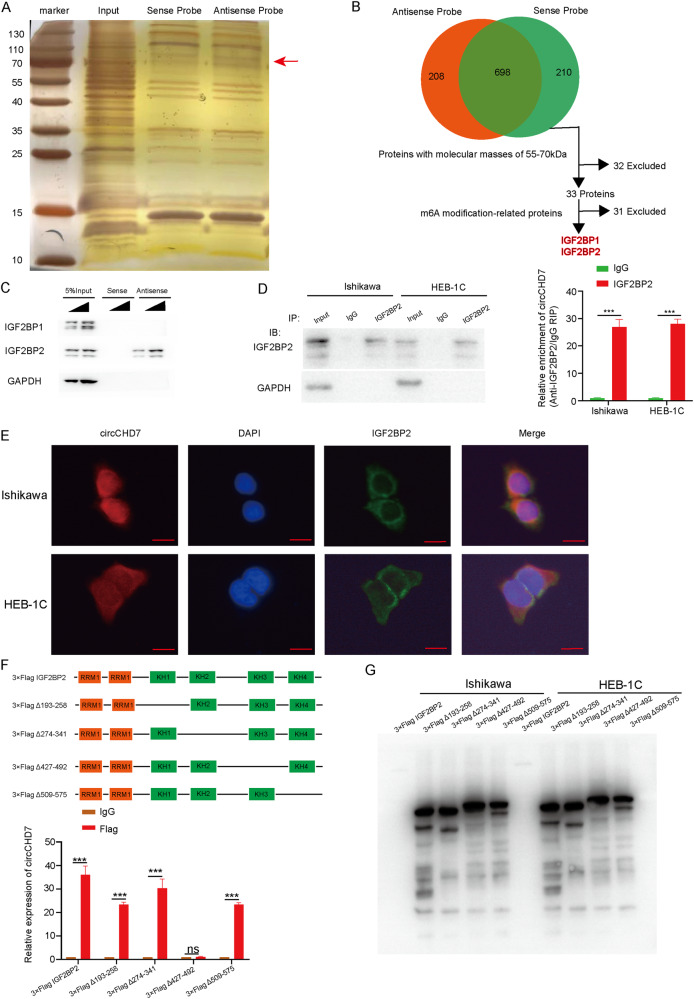
CircCHD7 interacts with IGF2BP2 proteins, located in the EC cytoplasm. **A** Silver staining revealed proteins pulled down by biotin-labelled circCHD7 antisense or sense probes in Ishikawa cell lysates. **B** Analyses of mass spectrometry assay were conducted to identify proteins that interact with circCHD7. **C** Western blot assay indicated that protein pulled down from Ishikawa and HEC-1B cells lysates by biotin-labelled circCHD7 antisense or sense probes. **D** RIP and qRT-PCR assays using a specific IGF2BP2 antibody showed an interaction between circCHD7 and IGF2BP2 in Ishikawa and HEC-1B cells. **E** Double RNA-FISH and immunofluorescence staining assays showed co-localization of circCHD7 (red) and IGF2BP2 (green) in Ishikawa and HEC-1B cells, and nuclei were stained with DAPI (blue). **F** Relative enrichment of circCHD7 levels was detected by qRT-PCR following forced expression of full-length or truncated Flag-tagged recombinant IGE2BP2 protein in Ishikawa cells. **G** Full-length or truncated Flag-tagged recombinant IGF2BP2 proteins were verified by western blot assays. Scale bar: 100 μm, *P < 0.05; **P < 0.01; ***P < 0.001.

Next, the specific mechanism of circCHD7 in EC progression was investigated. Many studies have shown that circRNAs could play a regulatory role by sponging miRNAs or binding proteins [27, 28]. In addition, the AGO2-RIP assay was performed to determine whether circCHD7 could sponge miRNAs. RNA immunoprecipitation assay (RIP) was conducted in Ishikawa cells and HEC-1B cells using an antibody specific for argonaute 2 (AGO2). The results showed no significant difference between the IgG and AGO2 groups, suggesting that circCHD7 may not act as a “miRNA sponge” (Supplementary Fig. 1J). Subsequently, circCHD7 was hypothesized to exert its role by binding with functional proteins. Proteins pulled down with a biotin-labeled circCHD7 antisense probe or sense probe were examined by western blot assay and silver staining (Fig. 3A). MS analysis (Supplementary Table 3) was then performed, and 65 different RBPs (RNA Bingding Proteins) were screened after overlapping the pulled-down proteins in Ishikawa cells. Two RBPs with protein molecular weight between 55–70 kDa, which were m^6^A modification-related proteins, were pulled down by biotin-labeled circCHD7 antisense probe in Ishikawa cells, including IGF2BP1 and IGF2BP2 (Fig. 3B). Moreover, the results of MS analysis showed that the AGO2 protein could not bind to circCHD7, which was consistent with the previous results. Biotin-labeled RNA pulldown experiments further showed that the circCHD7 antisense probe was able to interact dose-dependently with IGF2BP2 but not with IGF2BP1 in Ishikawa cells (Fig. 3C). The interaction between circCHD7 and IGF2BP2 was further validated through RIP assay (Fig. 3D). Furthermore, RNA-FISH and immunofluorescence assays demonstrated that circCHD7 and IGF2BP2 were co-localized in the cytoplasm of Ishikawa and HEC-1B cells (Fig. 3E).

Furthermore, the truncated structure of Flag-tagged recombinant IGF2BP2 proteins was constructed to identify the structural region of circCHD7 interacting with IGF2BP2. The results of the binding experiments revealed that deletion of the 427-492 amino acid region of the FLAG-tagged IGF2BP2 protein, but not the other structural domains, significantly abolished the interaction of circCHD7 with IGF2BP2, suggesting that the 427-492 amino acid region of IGF2BP2 played an essential role in its interaction with circCHD7 (Fig. 3F, G). These results indicate that circCHD7 could potentially interact with the IGF2BP2 protein in the EC cell cytoplasm.

### IGF2BP2 formed the circCHD7/IGF2BP2 complex by m^6^A modification in EC cells

**Fig. 4:**
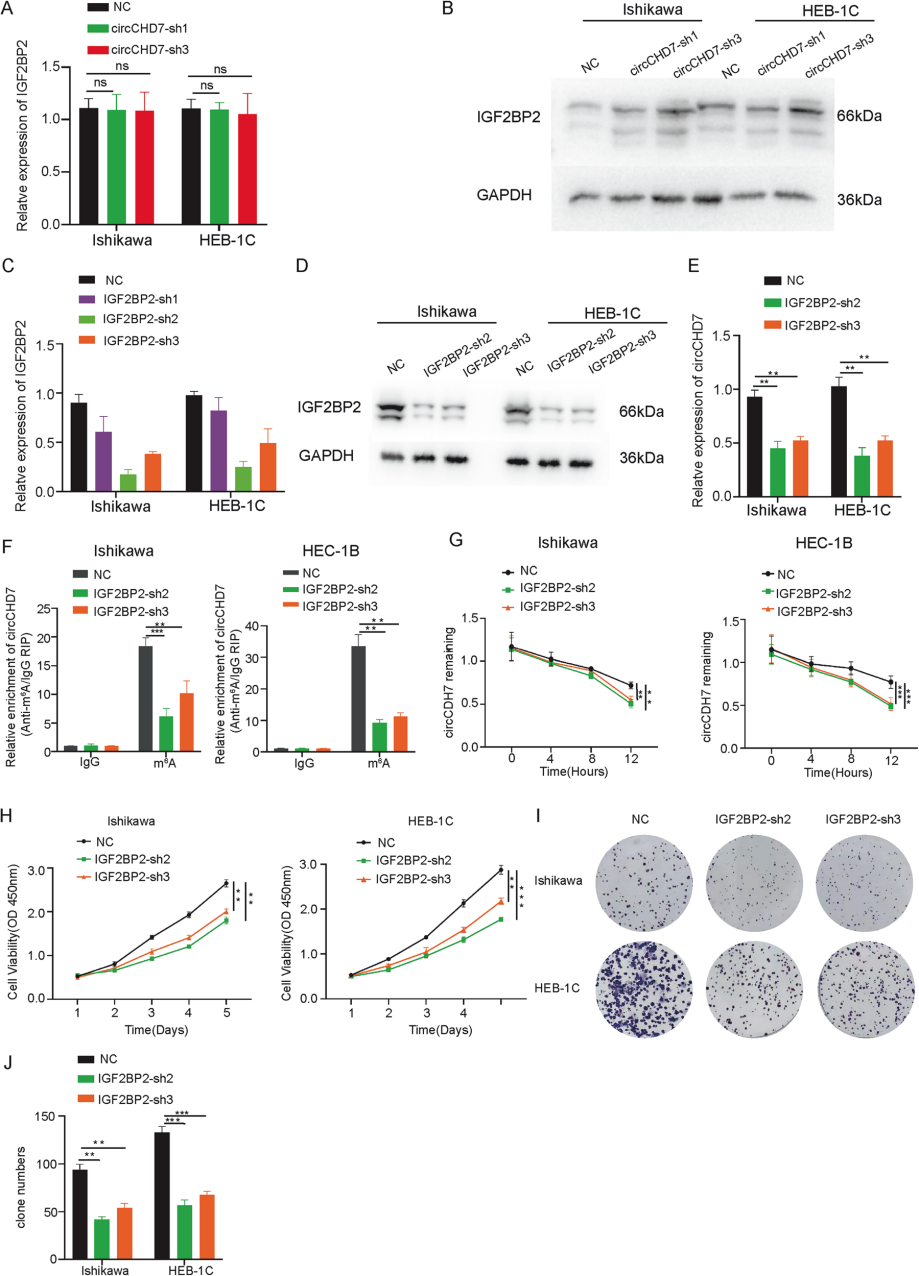
IGF2BP2 formed the circCHD7/IGF2BP2 complex by m^6^A modification in EC cells. **A**, **B** The expression levels of IGF2BP2 in Ishikawa cells and HEC-1B cells were detected by qRT-PCR and western blot assays upon circCHD7 knockdown. **C**, **D** The expression levels of IGF2BP2 were detected by qRT-PCR and western blot assay following IGF2BP2 knockdown in Ishikawa cells and HEC-1B cells. **E** The expression levels of circCHD7 were examined by qRT-PCR in Ishikawa cells and HEC-1B cells transfected with empty or sh-IGF2BP2 plasmid. **F** RIP-qPCR revealed the relative m^6^A modification levels of circCHD7 in Ishikawa cells and HEC-1B cells transfected with sh-IGF2BP2 plasmid or control. **G** Relative remaining RNA levels of circCHD7 were analysed by qRT-PCR in Ishikawa cells and HEC-1B cells transfected with sh-IGF2BP2 plasmid following treatment with actinomycin D at the indicated time points. **H** Viability of Ishikawa cells and HEC-1B cells was assessed at different times using CCK8 reagent. **I**, **J** Colony formation assay of Ishikawa cells and HEC-1B cells after transfection with sh-IGF2BP2 plasmid. *P < 0.05; **P < 0.01; ***P < 0.001.

To investigate further the interaction between circCHD7 and IGF2BP2, there was no effect on both mRNA and protein levels of IGF2BP2 in Ishikawa cells and HEC-1B cells following knockdown of circCHD7 Fig. 4A, B). Subsequently, IGF2BP2 knock-down Ishikawa cells and HEC-1B cells were constructed (Fig. 4C, D). However, the RNA levels of circCHD7 were suppressed after silencing IGF2BP2 (Fig. 4E). Considering that IGF2BP2 is an important component in the regulation of m^6^A methylation modifications, previous studies have reported that circRNAs could be regulated by the m^6^A reader IGF2BP2 [29]. Moreover, the MeRIP-PCR assay results revealed that m^6^A modification levels of circCHD7 in Ishikawa cells and HEC-1B cells were inhibited by IGF2BP2 knockdown (Fig. 4F). To determine the effect of IGF2BP2 on circCHD7 RNA stability, qRT-PCR was used to detect the level of circCHD7 in actinomycin D-treated EC cells at different times. The results suggested decreased stability of circCHD7 after silencing IGF2BP2 (Fig. 4G).

Previous studies have demonstrated that IGF2BP2 plays an oncogenic role in the development and progression of pancreatic cancer, breast cancer, and other diseases [30, 31]. However, the role of IGF2BP2 in EC has not yet been elucidated. Western blot, qRT-PCR assays, and IHC were performed on human EC specimens and normal endometrial tissues. IGF2BP2 was highly expressed in EC tissues compared with normal endometrial tissues, both at the protein and mRNA levels (Supplementary Fig. 2A–C). To elucidate the biological role of IGF2BP2 in promoting EC progression, the Ishikawa cells and HEB-1C cells were transfected with sh-IGF2BP2 or a negative control plasmid. Moreover, the CCK8, clone formation, and EdU assays revealed that the proliferative capacity of EC cells (Ishikawa cells and HEC-1B cells) was diminished following IGF2BP2 silencing (Fig. 4H–J, Supplementary Fig. 2D, E). These results demonstrate that IGF2BP2 exerts an important role in EC progression.

### The CircCHD7/IGF2BP2 complex enhances PDGFRB mRNA stability in EC cells

**Fig. 5:**
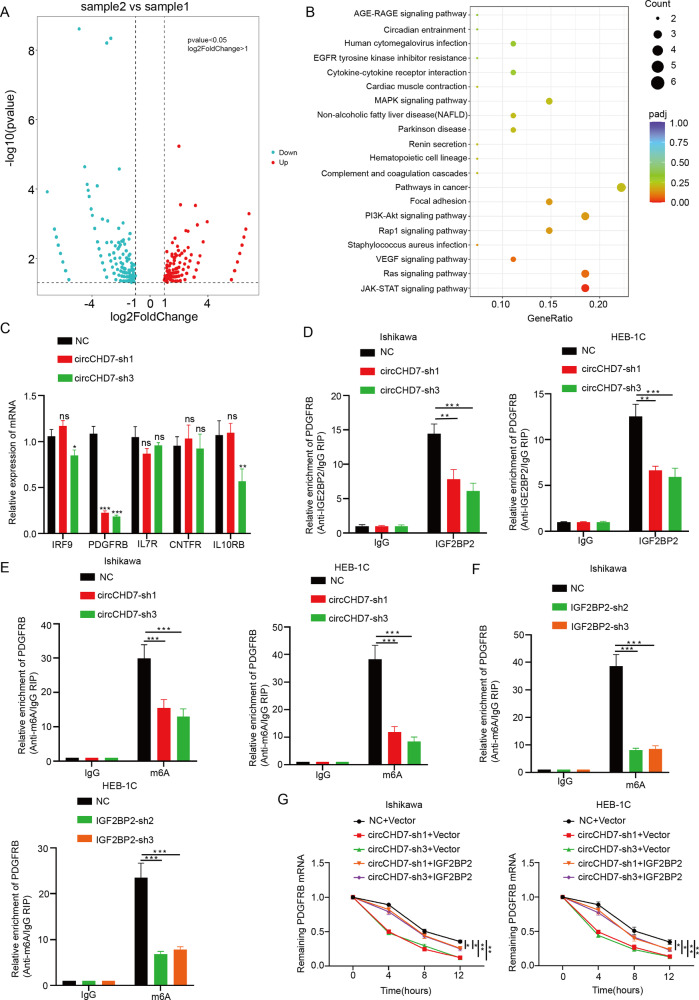
CircCHD7/IGF2BP2 complex enhances PDGFRB mRNA stability in EC cells. **A** Volcano diagram depicting differentially expressed mRNAs upon overexpression of circCHD7 in Ishikawa cells. **B** Kyoto Encyclopedia of Genes and Genomes (KEGG) analyses enrichment pathways of differentially expressed genes. **C** qRT-PCR indicated the expression levels of target gene mRNAs after transfection of sh-circCHD7 in Ishikawa cells. **D** RIP-qPCR revealed the level of PDGFRB mRNA bound to IGF2BP2 protein in Ishikawa and HEC-1B cells transfected with sh-circCHD7. **E** MeRIP-qPCR revealed the relative m^6^A modification levels of PDGFRB mRNA upon Ishikawa cells and HEC-1B cells transfected with sh-circCHD7. **F** MeRIP-qPCR revealed the relative m^6^A modification levels of PDGFRB mRNA upon Ishikawa cells and HEC-1B cells transfected with sh-IGF2BP2. **G** Relative remaining mRNA levels of PDGFRB in Ishikawa cells and HEC-1B cells transfected with control, sh-circCHD7 and co-transfected with null or IGF2BP2 were analyzed by qRT-PCR after treatment with actinomycin D at the indicated time points. ns > 0.05 *P < 0.05; **P < 0.01; ***P < 0.001.

To gain insight into the molecular function of circCHD7 in EC progression, an RNA sequence was employed to depict the gene expression profile of Ishikawa cells with circCHD7 overexpression. DESeq analyses were performed to identify differentially expressed genes relative to the control group, with filtering conditions |log_2_ FoldChange | >1 and *P* < 0.05. Among these, 257 genes were up-regulated and 290 genes were down-regulated in circCHD7 overexpressed cells (Fig. 5A, Supplementary Table 4). Then, the differential genes were subjected to Kyoto Encyclopedia of Genes and Genomes (KEGG) pathway enrichment analysis, revealing that upregulated genes were significantly enriched in the JAK/STAT signaling pathway (Fig. 5B). Furthermore, the JAK/STAT signaling pathway in the circCHD7 and vector mRNA-seq data results were combined, identifying 5 genes, namely IRF9, PDGFRB, IL7R, CNTFR, and IL10RB. Subsequently, the expression of these genes was explored. qRT-PCR analysis confirmed that the expression of PDGFRB was significantly reduced in sh-circCHD7-induced Ishikawa cells and HEC-IB cells compared to the other four genes (Fig. 5C).

IGF2BP2 is a novel established N6-methyladenosine (m^6^A) “reader” that can influence mRNA fate in an m^6^A-dependent manner. Subsequent experiments explored whether PDGFRB is regulated by circCHD7/IGF2BP2 complex in a m^6^A manner. RIP-qPCR assays showed that anti-IGF2BP2 antibodies precipitated PDGFRB mRNA and that circCHD7 knockdown significantly reduced PDGFRB mRNA enrichment (Fig. 5D). Furthermore, MeRIP-qPCR assays using the m^6^A antibodies revealed that METTL3, circCHD7, and IGF2BP2 knockdown resulted in significant down-regulation of PDGFRB mRNA levels in Ishikawa cells and HEC-1B cells (Supplementary Fig. 3A, Fig. 5E, F). In addition, RNA stability assays indicated that circCHD7 knockdown significantly diminished the stability of PDGFRB mRNA in Ishikawa cells and HEC-1B cells; however, overexpression of IGF2BP2 may reverse this effect (Fig. 5G). The expression levels of IGF2BP2 were detected by qRT-PCR and western blot assays in Ishikawa cells and HEC-1B cells of IGF2BP2 transfectants or corresponding vector cells (Supplementary Fig. 3B). Collectively, these results suggest that the circCHD7/IGF2BP2 complex enhances PDGFRB mRNA stability.

### CircCHD7 accelerates EC progression through the JAK/STAT signaling pathway

**Fig. 6:**
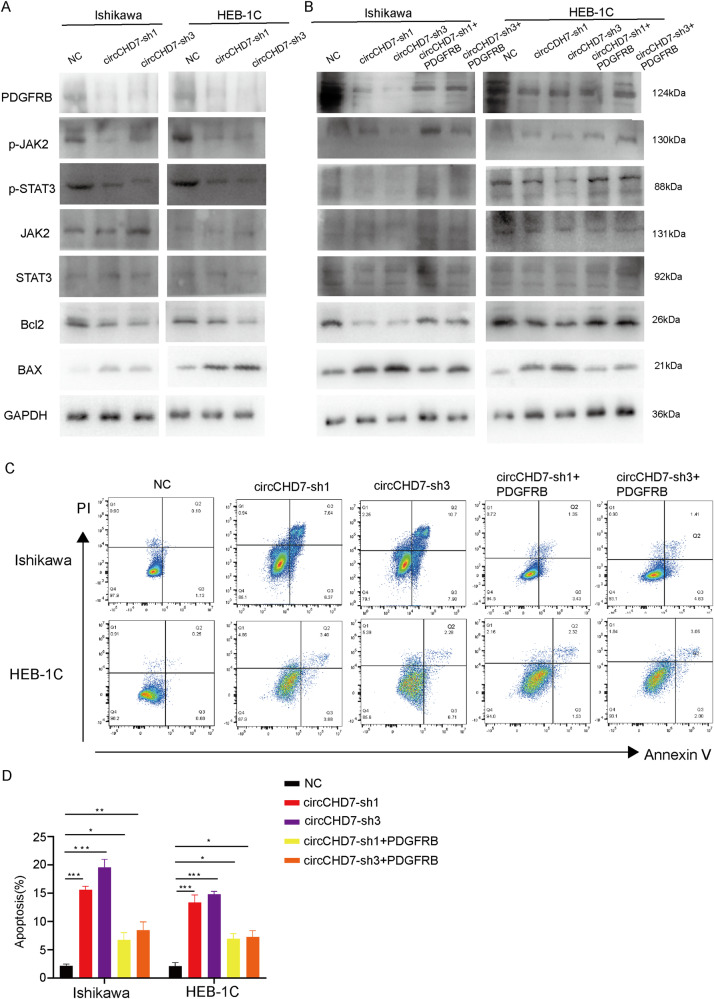
CircCHD7 accelerates EC progression through the JAK/STAT3 signaling pathway. **A** Western blot assays revealed the protein levels of PDGFRB, p-JAK2, p-STAT3, JAK2, STAT3, Bcl2, and BAX in Ishikawa cells and HEC-1B cells transfected with control or sh-circCHD7. **B** Western blot assays showed the protein levels of PDGFRB, p-JAK2, p-STAT3, JAK2, STAT3, Bcl2, and BAX transfected with control, sh-circCHD7 and co-transfected with null or PDGFRB in Ishikawa cells and HEC-1B cells. **C**, **D** Flow cytometry assay showed apoptosis rates of Ishikawa and HEC-1B cells transfected with control, sh-circCHD7 and co-transfected with null or PDGFRB. *P < 0.05; **P < 0.01; ***P < 0.001.

As previously described, RNA-seq was used in Ishikawa cells upon overexpression of circCHD7, and the differential genes were found to be mainly enriched in the JAK/STAT signaling pathway. Studies have shown that the JAK/STAT signaling pathway plays a critical role in endometrial cancer cell growth [32]. Moreover, the research [33] showed that inhibiting the PDGFR/JAK/STAT pathway significantly decreased proliferation in multiple novel patient-derived organoid models of endometrial cancer. The activation of this pathway was found to be a poor prognostic signal for the survival of patients with endometrial cancer from The Cancer Genome Atlas. Therefore, circCHD7 may act through the JAK/STAT signaling pathway. Western blot assays and cell flow experiments were performed to substantiate this hypothesis. At the protein level, phosphorylated JAK2 and STAT3 expressions were decreased following circCHD7 silencing in Ishikawa cells and HEC-1B cells; its related downstream genes, such as PDGFRB, Bcl2, and BAX, changed correspondingly, while JAK2 and STAT3 expressions remained unchanged. However, simultaneous transfection of sh-circCHD7 and overexpression of PDGFRB may counteract this effect (Fig. 6A, B). Ishikawa cells and HEC-1B cells were treated with the JAK inhibitor NSC 42834 to validate that circCHD7 affects endometrial cancer cell proliferation through the JAK/STAT signaling pathway. Western blot experiments results demonstrated that the JAK inhibitor NSC 42834 attenuated the pro-proliferative effects of circCHD7 on endometrial cancer cells (Supplementary Fig. 3C). Furthermore, cell flow experiments revealed that knockdown of circCHD7 induced apoptosis in Ishikawa cells and HEC-1B cells, while overexpression of PDGFRB partially reversed apoptosis (Fig. 6C, D). The PDGFRB levels were detected via qRT-PCR and western blot assays in Ishikawa cells and HEC-1B cells when transfected with PDGFRB or corresponding vector cells (Supplementary Fig. 3D). PDGFRB mRNA levels were detected in EC tissues and normal endometrial tissues by qRT-PCR, which suggested that the expression of PDGFRB was significantly higher in EC tissues compared to normal endometrial tissues (Supplementary Fig. 3E). Moreover, the expression of circCHD7 was positively correlated with the expression of PDGFRB in EC tissues (Supplementary Fig. 3F).

Altogether, these results demonstrated that circCHD7 could promote the proliferative activity by promoting PDGFRB expression through the JAK/STAT signaling pathway in EC cells.

### CircCHD7 enhances tumor growth in vivo

**Fig. 7:**
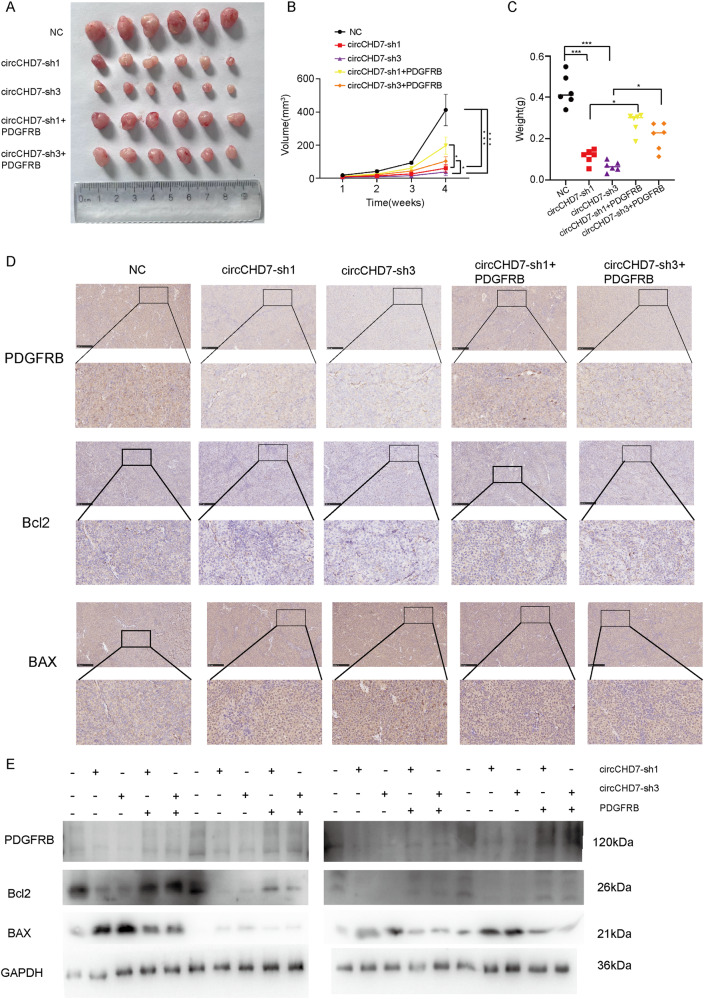
CircCHD7 enhances tumour growth in vivo. **A** Images of subcutaneous transplant tumours in BALB/c nude mice. **B**, **C** Tumour volume and weight measurement in BALB/c nude mice. **D** The relative protein levels of PDGFRB, Bcl2, and BAX were determined in subcutaneous xenograft tumours by IHC. **E** The expression of PDGFRB, Bcl2, and BAX in tumours were analyzed by western blot assay. Scar: 250 μm; *P < 0.05; **P < 0.01; ***P < 0.001.

To explore the relationship between circCHD7 and EC growth, Ishikawa cells stably transfected with sh-circCHD7, sh-NC vector, or both sh-circCHD7 and PDGFRB vectors were injected subcutaneously into BALB/c nude mice. The size and weight of the tumors were measured weekly. Supporting the results obtained in vitro, xenograft experiments showed that the size and weight of tumors in the sh-circCHD7 group were significantly smaller than those in the sh-NC group. However, overexpression of PDGFRB partially reversed this effect (Fig. 7A–C). These subcutaneous tumors were further assayed and subjected to immunohistochemical staining. The results showed that the protein levels of PDGFRB and Bcl2 were significantly decreased in the sh-circCHD7 group compared with the sh-NC group, while the protein level of BAX was significantly elevated. These differences were partially reversed by co-transfection with the PDGFRB overexpression vector. The results of western blot assays were consistent with the results of immunohistochemistry, as described above (Fig. 7D, E). Collectively, these results revealed that circCHD7 is an oncogene associated with the proliferation of EC cells.

**Fig. 8:**
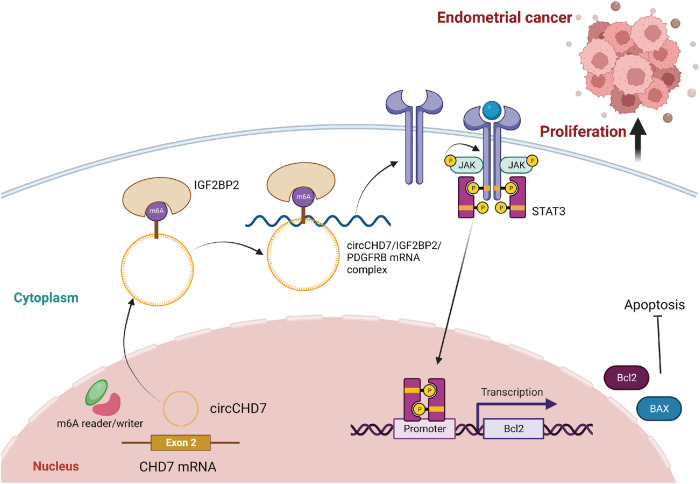
m6A-modified circCHD7 promotes EC progression by enhancing PDGFRB mRNA stability and activating JAK/STAT signaling pathway via interacting IGF2BP2.

Therefore, m6A-modified circCHD7 promotes EC progression by enhancing PDGFRB mRNA stability and activating JAK/STAT signaling pathway via interacting IGF2BP2 (Fig. 8).

## Discussion

With the advancement of biochemical enrichment strategies and deep sequencing, abundant evolutionarily conserved circRNAs have been identified in mammalian cells and tissues; however, the biogenesis of circRNAs remains relatively obscure [6, 34]. Previous studies have shown that circRNAs play a variety of important roles in cellular physiological processes as miRNA sponges, RBP-binding molecules, transcriptional regulators, protein backbones, or protein translation templates [35]. In previous studies, we have shown that circESRP1 can act as a “miRNA sponge” to regulate EC progression [36]; CircRAPGEF5 interacts with the RNA-binding protein RBFOX2 and promotes ferroptosis resistance in EC cells by regulating the selective splicing of TFRC [37]. Research is being carried out to elucidate the role of m^6^A in EC. Considering the role of IGE2BP2 in EC, IGF2BP2 function was associated with circRNAs to explore the possibility of m^6^A-circRNA interactions. In the present study, circCHD7 could interact with the key m^6^A-binding protein IGF2BP2 and mediate m^6^A modification of downstream target mRNAs. These findings illustrate for the first time in EC that circRNAs can bind to key m^6^A-binding proteins (reader) and reveal a novel role for circRNAs in regulating mRNA m^6^A modification.

With a unique covalent closed-loop structure and a specific tertiary structure, circRNAs play crucial roles in circRNA-protein interactions. In addition, m^6^A has been shown to be a substantial transcriptionally relevant modification in both mRNAs and ncRNAs, including circRNAs. This broadly involves post-transcriptionally associated mRNAs. However, the mechanisms underlying the effects of m^6^A modifications on biological aspects of cellular circRNAs remain unclear. Previous studies have shown that circCCDC134 enhanced its stability and promoted HIF1A transcription by m^6^A modification, thereby promoting cervical cancer growth and metastasis by serving as a recruitment p65 in the nucleus and a miR-503-5p sponge regulating MYB expression in the cytoplasm [38]. M^6^A-modified circQSOX1 promoted colorectal cancer tumorigenesis by promoting PGAM1 expression by sponging miR-326 and miR-330-5p and further facilitated colorectal immune escape by activating glycolysis and inactivating anti-CTLA-4 therapeutic response [39]. CircNSUN2 was exported from the nucleus to the cytoplasm by the YTHDC1 protein in a m^6^A methylation-dependent manner. Increased circNSUN2 interaction with IGF2BP2 in the cytoplasm enhanced the stability of HMGA2 mRNA, leading to increased aggressiveness of colorectal cells [40]. In this study, circCHD7 interacted with the m^6^A binding protein IGF2BP2 and regulated its stability. Notably, circCHD7 was found to bind to the 427-492 amino acid regions of the KH domain of the IGF2BP2 protein, promoting m^6^A modification of PDGFRB mRNA. Therefore, the binding of circRNAs to RBPs might alter these proteins’ spatial distance, expose or cover up their active site, and might transform their spatial conformation, thus affecting the interaction between RBP and other proteins.

Platelet-derived growth factor receptor beta (PDGFRB) is a kind of tyrosine protein kinase, acting as a cell surface receptor for homodimers PDGFB and PDGFD and heterodimers formed by PDGFA and PDGFB [41, 42]. Studies have shown that PDGFRB plays an essential role in the regulation of tumor proliferation, apoptosis, differentiation, drug resistance, and invasion [43, 44]. Previous studies have suggested that PDGFRB may be associated with the development of EC [45]; yet, the specific mechanism is unclear. The present study revealed that overexpression of PDGFRB partially reversed the reduced apoptosis rate due to the knockdown of circCHD7. Furthermore, circCHD7 interacted with IGF2BP2 to facilitate PDGFRB expression in an m^6^A-dependent fashion by enhancing the stability of PDGFRB mRNA. These further provide strong arguments for post-transcriptional regulation of PDGFRB. The m^6^A demethylation mediated by FTO relies on its m^6^A RNA demethylase activity to activate the PDGFRB/ERK signaling axis, playing an oncogenic role in NPM1-mutant AML and providing new epigenetic insights into the future treatment of this distinct aleukaemic entity [46]. Emerging research evidence suggests that PDGFRB activates the JAK2/STAT3 signaling pathway to play a regulatory role [47, 48]. The JAK/STAT pathway is a highly conserved signal transduction pathway that mediates downstream events such as hematopoiesis, immune adaptation, tissue repair, inflammation, apoptosis, and adipogenesis [49]. IL-6 secreted by tumor fibroblasts induces activation of the Janus kinase/signal transducer and activator of transcription (JAK/STAT) pathway in tumor cells, which promotes the expression of c-Myc, thereby facilitating EC cell proliferation in vitro and in vivo [32]. In the current study, m^6^A-modified circCHD7 interacted with IGF2BP2 to enhance the stability of PDGFRB mRNA, which activated the JAK/STAT signaling pathway and promoted EC cell proliferation. However, the specific site of circCHD7/IGE2BP2-mediated m^6^A modification of PDGFRB and whether it regulates EC chemosensitivity remains to be further clarified.

## Conclusion

In summary, this study demonstrates that circCHD7, a novel circRNA, is upregulated in EC cells. Biologically, circCHD7 promotes tumor cell proliferation and suppresses apoptosis. Mechanistically, m^6^A-modified circCHD7 binds to IGF2BP2 and activates the JAK/STAT signaling pathway by enhancing mRNA stability to promote the expression of the target gene PDGFRB in an m^6^A-dependent manner. Our study unveils that the m^6^A-circRNA approach targeting the circCHD7/IGF2BP2/PDGFRB pathway is a potential strategy to inhibit EC progression.

### Supplementary information

Supplementary Figure

Supplementary Figure Legend

Supplementary Table 1

Supplementary Table 2

Supplementary Table 3

Supplementary Table 4

## Data Availability

The data analyzed in this study are included in the manuscript.
