# Supplementary figures and images for: IGF2BP2-modified circular RNA circCHD7 promotes endometrial cancer progression via stabilizing PDGFRB and activating JAK/STAT signaling pathway
Source: Cancer Gene Ther. 2024 May 22;31(8):1221–36. doi: 10.1038/s41417-024-00781-9 (PMC11327104; doi:10.1038/s41417-024-00781-9)

Supplementary Figure 1

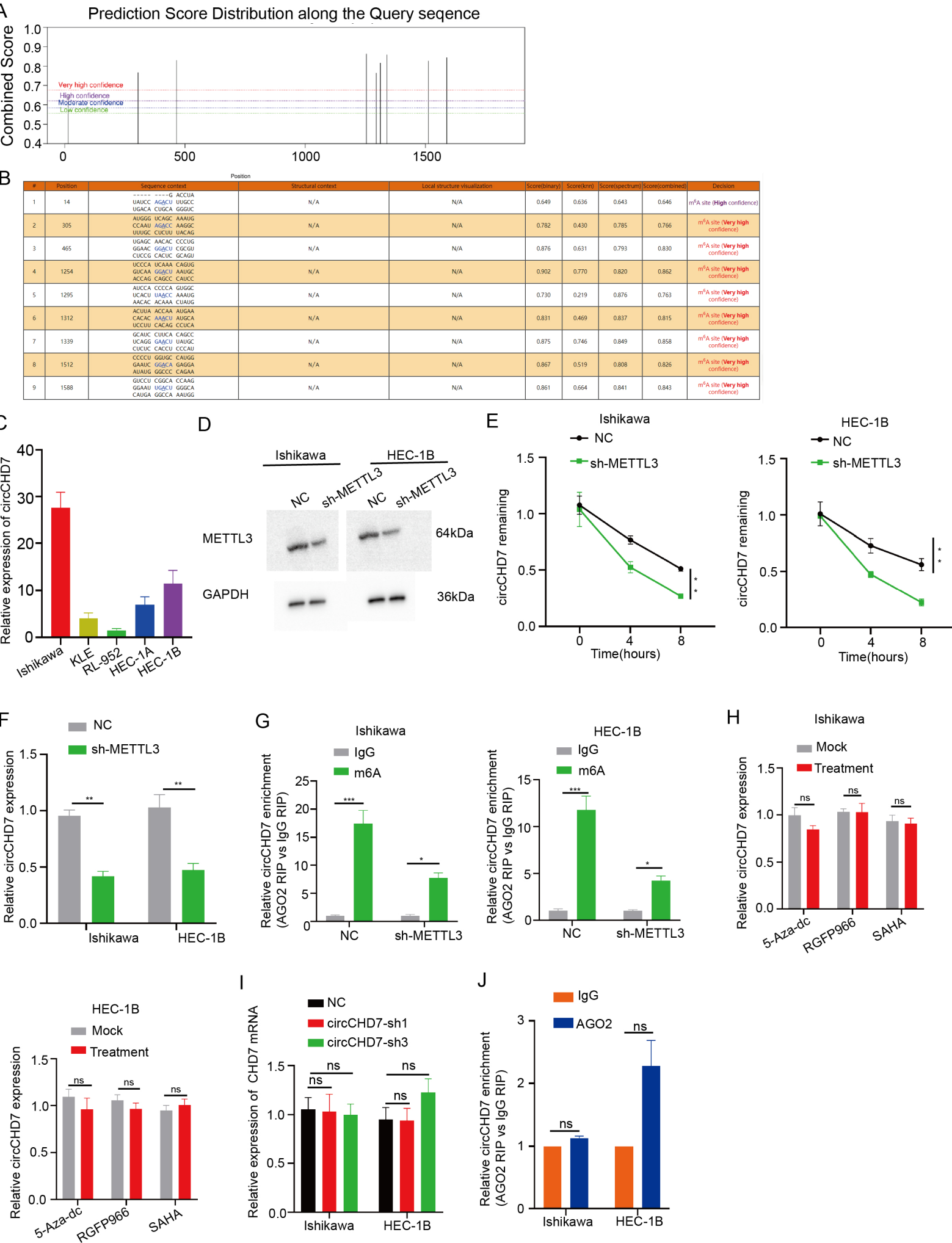

Supplementary Figure 2

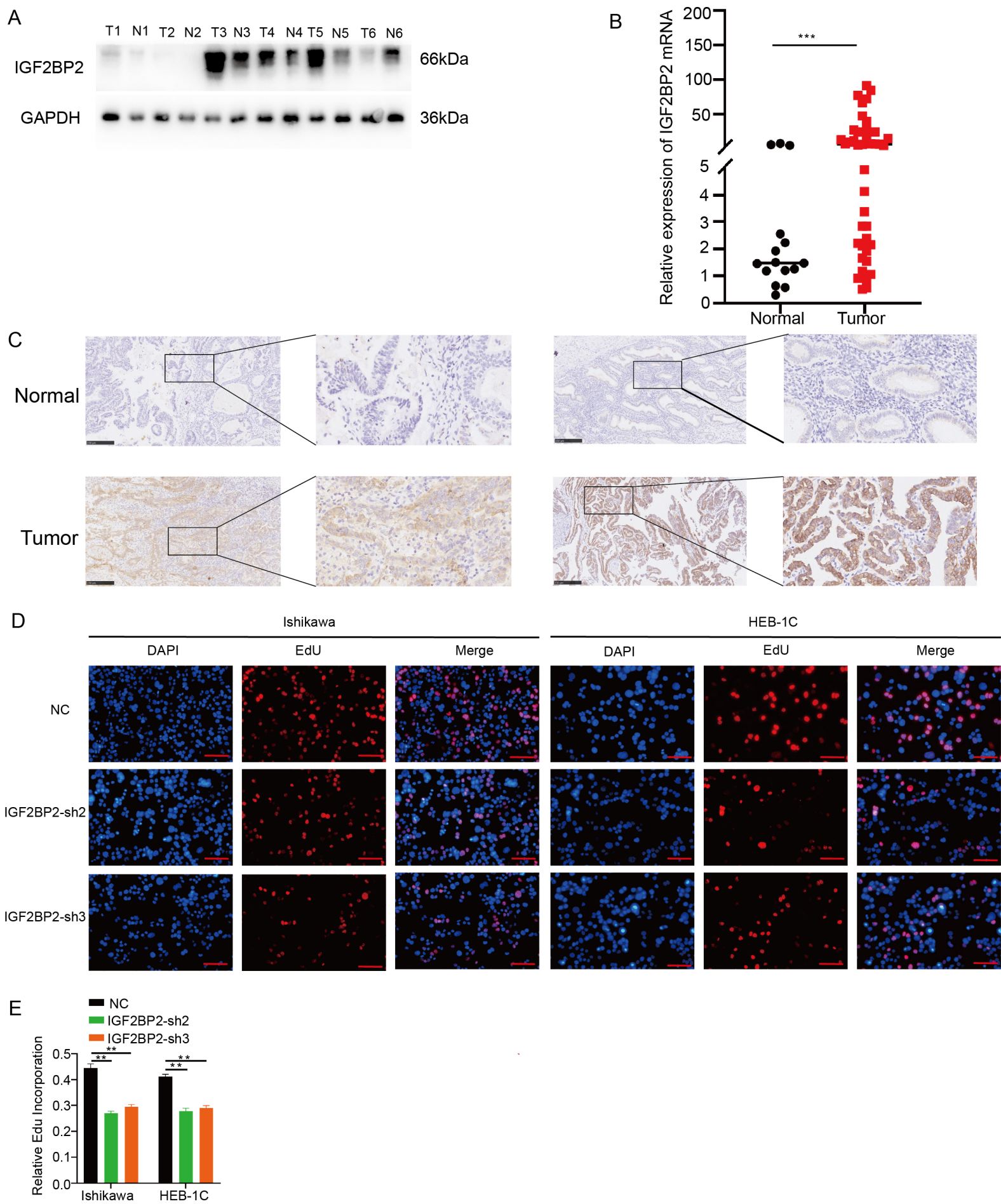

Supplementary Figure 3

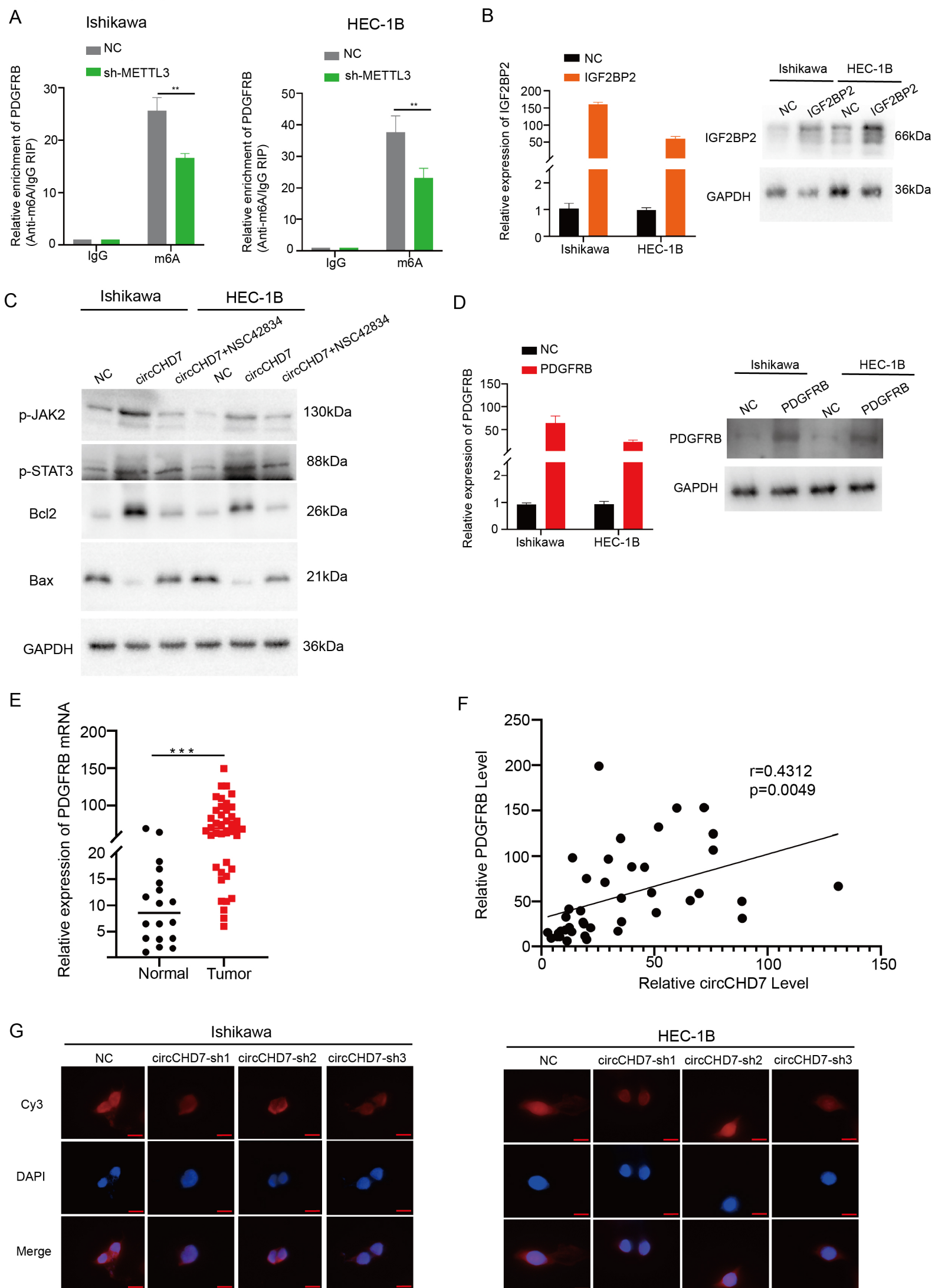

Supplement: Supplementary file 1 — Supplementary Figure [file 41417_2024_781_MOESM1_ESM.pdf]
