# Supplementary material for: IGF2BP2-modified circular RNA circCHD7 promotes endometrial cancer progression via stabilizing PDGFRB and activating JAK/STAT signaling pathway
Source: Cancer Gene Ther. 2024 May 22;31(8):1221–36. doi: 10.1038/s41417-024-00781-9 (PMC11327104; doi:10.1038/s41417-024-00781-9)
Supplement: Supplementary file 2 — Supplementary Figure Legend [file 41417_2024_781_MOESM2_ESM.docx]

**Supplementary Figure1** A-B SRAMP online database predicted m6A methylation modifications of circCHD7. C The relative expressions of circCHD7 in EC cell lines were analyzed by qRT-PCR. D The protein levels of METTL3 in Ishikawa and HEC-1B cells transfected with sh-METTL3 and the corresponding control were detected by western blot. E qRT-PCR assays were performed to detect the stability of circCHD7 when treated with actinomycin D. F The expression level of circCHD7 in Ishikawa and HEC-1B cells transfected with sh-METTL3 and the corresponding control were detected by qRT-PCR. G MeRIP-PCR was performed to detect the circCHD7 levels in m6A antibody-immunoprecipitated RNA compared to IgG-precipitated RNA. H qRT-PCR assays were performed to detect the stability of circCHD7. I The mRNA level of linear CHD7 was detected by qRT-PCR assay in Ishikawa and HEC-1B cells transfected with circCHD7-sh1, circCHD7-sh3, and the corresponding control. J Anti-AGO2 RIP assay was performed in Ishikawa cells and HEC-1B cells, followed by qRT-PCR to detect the capacity for AGO2 enrichment on circCHD7 compared to IgG. ns p > 0.05; * P < 0.05; ** P < 0.01; *** P < 0.001.

**Supplementary Figure2** A-B The relative expression levels of IGF2BP2 were determined in EC tissues and normal endometrial tissues by qRT-PCR and western blot assay. C The relative protein levels of IGF2BP2 were detected by IHC in EC tissues. D-F DNA synthesis in Ishikawa cells and HEC-1B cells was measured by Edu assay. Scar: 250μm，100μm; * P < 0.05; ** P < 0.01; *** P < 0.001.

**Supplementary Figure3** A MeRIP-qPCR revealed the relative m^6^A modification levels of PDGFRB mRNA upon Ishikawa cells and HEC-1B cells transfected with sh-METTL3. B The expression levels of IGF2BP2 were examined by qRT-PCR and western blot assay in Ishikawa cells and HEC-1B cells transfected with empty or IGF2BP2. C The relative proteins expressions were detected by western blot when Ishikawa cells and HEC-1B cells were treated with the JAK inhibitor NSC 42834. D The expression levels of PDGFRB were determined in Ishikawa cells and HEC-1B cells transfected with empty or PDGFRB by qRT-PCR and western blot assay. E The qRT-PCR was peformed to detect the PDGFRB mRNA levels of EC tissues. F The correlation between the transcript levels of circCHD7 and PDGFRB in EC tissues was analyzed. G FISH assay was to verify the specificity of circCHD7 fluorescence signaling in Ishikawa cells and HEC-1B cells transfected with circCHD7-sh1, circCHD7-sh2, circCHD7-sh3, and the corresponding control. Scar: 250μm; * P < 0.05; ** P < 0.01; *** P < 0.001.
